# Optimizing Trichiasis Case Finding to Attain the Elimination of Trachoma as a Public Health Problem

**DOI:** 10.3390/tropicalmed9070157

**Published:** 2024-07-11

**Authors:** Joy Shu’aibu, Grace Ajege, Caleb Mpyet, Michael Dejene, Sunday Isiyaku, Abubakar Tafida, Michaela Kelly, Innocent Emereuwa, Paul Courtright

**Affiliations:** 1 Sightsavers, Abuja 900231, Nigeria; 2 Department of Family Medicine, Bingham University, Karu 961105, Nigeria; 3 Department of Ophthalmology, University of Jos, Jos 930003, Nigeria; 4 Public Health Consultancy Services, Addis Ababa 1169, Ethiopia; 5 Ministry of Health Jigawa State, Dutse 720222, Nigeria; 6 Sightsavers, West Sussex RH16 4BX, UK; 7 Health and Development Support Programme, Jos 930003, Nigeria; 8 Kilimanjaro Centre for Community Ophthalmology, Division of Ophthalmology, University of Cape Town, Cape Town 7925, South Africa

**Keywords:** trachomatous trichiasis, case finding, gender, Nigeria

## Abstract

Background: As national trachoma programmes increase efforts to reduce the burden of trachomatous trichiasis (TT), TT case finding and referral are critical public health programme components. Our research aimed to explore the most effective and efficient approaches to finding, referring, and managing TT cases. Methods: This was a prospective descriptive study, utilizing both routine programme data and primary data collection. This study compared four different approaches to finding TT cases across three different local government areas (LGAs) in Kano State, Nigeria. Each of the study LGAs was divided into four sub-units to accommodate the four different approaches. Results: The number of outreach attendees was 4795 across the four case finding approaches, and this varied hugely, with the smallest number and proportion (403, 0.26%) in settings only employing house-to-house case finding and the largest number and proportion (1901, 0.99%) when town criers were used. That said, the proportion of TT cases among people presenting at outreach was highest (32.5%) when house-to-house case finding was used and lowest (10.3%) when town criers were used. More female TT patients were found (53–70%) and had surgery (79–85%) compared to male cases, across all approaches. The average project expenditure for finding one TT case was similar for approaches that included house-to-house case finding (USD 5.4–6.3), while it was 3.5 times higher (USD 21.5 per TT case found) when town criers were used. Discussion: This study found that the house-to-house TT case finding approaches were the most efficient method with the highest yield of TT cases. Including other eye condition and/or vision testing yielded similar results but required more personnel and cost.

## 1. Introduction

Trachoma is the leading infectious cause of blindness, affecting the most marginalized and disadvantaged populations in endemic countries where it is prevalent [1]. Blindness from trachoma results from damage to the cornea from constant rubbing by in-turned eye lashes (called trachomatous trichiasis—TT). This results from recurrent conjunctival inflammation due to trachoma infection [2]. Research has shown that women are at a higher risk for developing TT compared to men [3,4]. The economic impact of trachoma on individuals and communities is high [5,6].

The National Survey of Blindness and Low Vision conducted in Nigeria in 2005–2007 found trachoma to be responsible for 8.3% of blindness in the northern savannah belt of the country [7]. Kano State in northern Nigeria is trachoma-endemic, and in 2013, there were an estimated 47,988 people living with TT [8].

In 1998, the World Health Assembly passed a resolution calling for the global elimination of trachoma as a public health problem by 2020 [9]. The elimination target was recently revised to 2030 [10]. Nigeria has proposed to reach elimination by the year 2030. The WHO integrated strategy to achieve trachoma elimination, the SAFE strategy [11], consists of surgery to correct TT, antibiotics to treat chlamydial infection, and facial cleanliness and environmental improvements to interrupt the transmission of trachoma.

Although the surgical management of TT has been shown to be cost-effective, finding TT cases continues to be challenging, and the success of any trachoma programme is dependent on the effectiveness of finding TT cases [12] and ensuring that they are offered management, often at a surgical outreach [13]. Using routinely collected programme data, previous research has suggested that house-to-house TT case finding is more effective and efficient and is more likely to reach women compared to other, non-focused mobilization approaches [14].

This study was carried out to understand which TT case finding approach is most effective and efficient and how different approaches to community mobilization can be implemented in trachoma-endemic settings in Nigeria.

## 2. Methods

This study was conducted in 2017 in Kano State in northern Nigeria. The state has 44 local government areas (LGAs) and a population of about 9 million people [15]. The majority of the population are settled agriculturists and herdsmen; however, there are also pastoral Fulani, some of whom are nomadic. This study was carried out in three LGAs in Kano State, Kura, Ajingi, and Tudun Wada, where there was ongoing support for trachoma elimination activities and where TT surgery activities were due to be commenced. In 2013, the age–sex-adjusted prevalence of TT in adults (age 15+) in Kura was 0.71% (95% CI 0.35–1.25), in Ajingi was 2.24% (95% CI 1.42–3.49), and in Tudun Wada was 2.21% (95% CI 1.47–3.07) [8].

This was a prospective implementation study of four different approaches to TT case finding. It used routinely collected programme data in addition to data collected during outreach and during case finding. The three LGAs were geographically distant from each other, and each LGA was divided randomly into four sub-units corresponding to the four case finding approaches.

**House-to-house case finding for TT by community volunteers (approach #1).** Following training, case finders (CFs) went to all houses in their respective communities, examined all adults for TT, and referred all suspected TT patients to a pre-set outreach where case management was available [13]. The outreach took place two weeks after the commencement of house-to-house case finding. At the outreach, only TT case management was provided, but referrals were provided for patients with other eye diseases, while minor eye ailments requiring tetracycline eye ointment (TEO) were addressed and documented.

Prior to case finding, a map of the area was used to ensure that all communities (including migrant populations) in the area were included. A systematic plan was developed for visiting each community and which locations will be used for outreach to ensure reasonable access to the outreach site by all residents. TT case finders were trained for half a day using the International Coalition of Trachoma Control (ICTC) manual [12] to find suspected TT cases. They were provided a torch to assist with case detection [16]. Each case finder was assigned a specific number of houses to visit, to register all suspected TT cases found over two weeks of case finding, and to inform cases to come to the outreach location on the pre-set day. A day prior to the outreach, the number of suspected cases on each case finder’s register was communicated to the project team and the outreach location and day confirmed. The necessary number of human resources and days were calculated from the number of patients expected at each outreach. Case finders were encouraged to accompany their suspected cases to the outreach, which was conducted according to the ICTC trichiasis surgical outreach manual [13]. The outreach team examined all people who attended; any person with a condition other than TT was referred for treatment and their details documented. TT cases were managed appropriately.

**House-to-house case finding for TT by community volunteers plus management of other eye conditions at outreach (approach #2).** All steps undertaken for house-to-house case finding were the same as described in the first method. The only difference was that outreach included the treatment of common eye conditions, such as conjunctivitis and ocular allergy. The TT outreach team tested the visual acuity (VA) of all people who presented, and anyone with visual impairment (visual acuity of <6/18) was examined by an ophthalmic nurse (not the TT surgeon) and treated accordingly or booked for surgery at the nearest suitable location if an operable cataract was detected.

**House-to-house case finding for TT and testing of visual acuity by a community health worker (approach #3).** Community health workers, rather than community volunteers, were trained to examine for TT and to measure VA to detect if an individual eye had visual impairment (VI). They were provided a torch to assist with the detection of TT. The steps to ensure complete coverage of the areas were the same as in the previous methods. The one-day training included the detection, recording, and referral of TT and VI to a pre-set outreach. Case finders were requested to distinguish clearly on their register suspected TT cases and cases with suspected VI or both. At the outreach, the team tested the VA of all people who attended. Individuals with VI were examined by an ophthalmic nurse and treated accordingly or booked for surgery at the nearest suitable location if it was an operable cataract.

**Town criers announced an upcoming eye care outreach (approach #4).** Town announcers were identified and given a standardised message to advertise eye care services, including for TT, up to one week before and on the day of the outreach. As noted with methods #2 and #3, the outreach team tested VA, and persons with VI were examined separately, basic treatment was provided, and referrals were made, as indicated. In the absence of case finding, no data were available on suspected cases prior to outreach.

Each LGA was sub-divided, and sub-units were randomly allocated to one of the case finding approaches. All four approaches tested in this study were deployed in all three LGAs of study but at different times and in different sub-units. To reduce the risk of contamination, during advocacy and community entry, one of the key points emphasised was that interventions will be provided to the entire LGA in a cascaded approach, and individuals were encouraged to remain in their locations and await the intervention. Those presenting for care self-reported that they resided within the study cluster, but this was not verifiable.

The primary outcome measures were the number and proportion of TT cases identified and managed with each of the approaches tested. Findings were combined for all 3 LGAs for each approach. The cost of finding one TT case was a secondary outcome; this was derived from the total cost of allowance paid to case finders, the cost of case finder training, the cost of training town criers and their allowance, and allowances for the outreach team.

The data collected from each study site were entered into a Microsoft Excel spreadsheet and cleaned. The data were consolidated across the sites and analysed using the Statistical Package for Social Sciences (SPSS) software version 21. Odds ratios and 95% confidence intervals were calculated to assess associations.

All data collected were anonymized, and the confidentiality of patient information was ensured throughout this study. All data were stored in secure password-protected computers accessed by the research team only. All participants attending the outreach gave their informed consent to use their data for the purpose of this study. All TT cases presenting to outreach received appropriate treatment at no cost to them. The study protocol received ethical approval from the Institutional Review Boards of the Jos University Teaching Hospital and the Kano State Ministry of Health.

## 3. Results

Among the three approaches that used TT case finders, the number of suspected TT cases on case finder registers per population was highest in the approach (#3) that used community health workers rather than community volunteers; however, this approach had a low proportion of confirmed TT cases among those suspected to have TT (Table 1). In all three approaches, less than a half of the suspected cases presented to outreach, although suspected TT cases in areas using approaches #1 and #2, respectively, were 1.91 (95% CI 1.59–2.29, *p* < 0.001) and 1.45 (95% CI 1.26–1.68, *p* < 0.001) times less likely to present to outreach compared to approach #3. Among those that did present to outreach, the volunteer case finders (approach #1) had the largest proportion (46.1%) of confirmed TT cases.

Women accounted for 53.5–70% of people presenting to the outreach, highest among approach #1 (Table 2). Women accounted for 79.4–85.2% of TT cases operated, not statistically different between the approaches. Overall, there were 1.45 women to men presenting to outreach and 4.92 women to men having surgery (*p* < 0.001). Among the total number of people presenting to outreach, between 10.3% and 32.5% were confirmed to have TT, the lowest percentage in the approach using community criers rather than case finders (Table 2). Among confirmed TT cases, male and female, the uptake of surgery was high for all approaches. Gender-disaggregated information was not available on the CF register of suspected TT cases and hence not available for further analysis.

As expected, people self-presented to outreach, with the proportion ranging from 40 to 55% of outreach attendees. Among self-presenters, TT was relatively rare, ranging from 5.1% to 14.4% (Table 3). In the community crier approach 1901 people presented to outreach, but only 10.3% of them had TT. People presenting to outreach in areas using approach #1 were 4.21 times (95% CI 3.26–5.44, *p* < 0.001) more likely to have confirmed TT compared to the people in approach #4 (Table 3).

Combining the three house-to-house case finding approaches revealed that there were 2894 attendees at outreach, among whom 472 (16.3%) were TT cases, while using town criers, there were 1901 attendees at outreach, among whom 195 (10.3%) were TT cases (*p* < 0.001).

## 4. Discussion

This study compared four different approaches to case finding and referral to TT outreach. The findings are similar to recent research using routinely collected data [14]. The current study tested different approaches, and the comparisons observed should assist programme staff in making evidence-based decisions regarding TT case finding and outreach. This should lead to a more rapid scale-up of case finding, a key component to achieving the elimination of TT as a public health problem.

Our findings suggest that house-to-house case finding, whether combined with other eye care interventions or not, is much more efficient in ensuring that TT cases receive appropriate management, compared to community mobilization through town criers. Among the three case finding approaches used, community health workers identified significantly more suspected cases per population than volunteer case finders. The community health workers also had a significantly higher proportion of their suspected cases attend outreach. These two findings might suggest that community health workers, trained to recognize both TT and visual impairment, would be the best approach to case finding. That said, among the three case finding approaches, the volunteer case finders had the highest proportion of suspected TT cases confirmed to have TT. While we do not have an explanation, it is possible that community health workers listed people with other eye conditions as having TT. It is also possible that community health workers focused more effort on the testing of VA instead of examining for TT. It is not uncommon in areas with minimal eye care services for health workers to be pressured to list people with other conditions in the hope that they will receive a service.

Community volunteers have been effectively used as TT case finders in Egypt [17] and Sudan [18]. There are advantages in using community volunteers as TT case finders. First, the community has ownership of the project, as community leaders are involved in the selection of case finders. Second, community volunteers are more likely to know persons suffering from TT, as they live in the community and interact with them daily. Finally, since community volunteers are only trained to identify and record TT patients, they may be more focused. A randomized controlled trial in Tanzania showed that community treatment assistants undertaking a half-day training on finding TT cases identified five times more TT patients than community treatment assistants trained in the usual MDA package with a 30 min overview of TT [19].

It is well recognized that women bear a greater burden of TT compared to men [3,4]. Multivariate analysis of the baseline trachoma surveys in Kano State in 2013 revealed that women have a 3.3-fold (95% CI 2.9–3.7) higher risk of TT compared to men [8]. There are multiple reasons for why women have excess TT compared to men [3]. Programme managers need to consider the best approaches to reaching women with TT, first with case finding, counselling, support to attend outreach, surgery, etc. In our study, women outnumbered men as outreach attendees by 1.45:1, but this is considerably lower than the expected ratio of women to men for TT. We do not have information on the gender distribution of suspected TT cases referred by case finders, limiting our ability to assess whether one approach to case finding is superior to another for reaching women. That said, the ratio of women to men among surgical cases (4.92:1) matches the expected case ratio. Similar to research from Vietnam and Tanzania, the high uptake of surgery by all confirmed TT cases, regardless of case finding approach, is likely a function of the outreach team [20]. Good-quality counselling, high-quality surgery, and other factors encourage the acceptance of surgical management.

The high proportion of suspected TT cases not attending outreach has been noted before [21]. Community health workers appear to have been more successful in encouraging suspected TT cases to attend outreach compared to volunteers; nevertheless, considerable work is needed to ensure that suspected TT cases are examined by eye care workers and provided management. It should be noted that the findings from this study (2017) and other research led to considerable changes in programmes that have led to a significant reduction in loss to outreach in Nigeria.

Outreach, by its very nature, will end up including attendees who were not part of case finding efforts. In settings with limited eye care services, residents will learn of an upcoming outreach and attend if they have a felt need, regardless of the presence of TT. Thus, it was not surprising that just under half of those attending outreach were self-presenting. The efficiency of a TT programme is driven by the case finding approaches undertaken, the personnel required for outreach, and the prevalence of TT in the area. Managing large numbers of people at outreach is often difficult for a team, which often leads to adding more people to deal with the attendees. Thus, it is not surprising that the approach using town criers, which results in large numbers of attendees, but few TT cases, is three times more expensive per TT case, as more outreach staff are required. As TT becomes rarer, the number and proportion of TT cases will decrease, making the use of town criers even more inefficient.

As noted above, we are limited in our interpretation by the absence of information on gender among cases of suspected TT. One case finding approach may be more effective at reaching women than another. We have no information on the sub-unit prevalence of TT, and it is possible that there are focal differences which may have accounted for some of the variation in suspected TT cases. While we have the cost of case finding and outreach for TT, it is not possible to calculate the cost of the management of other eye conditions detected and managed through the different approaches.

## 5. Conclusions

In conclusion, this study suggests that house to house TT case finding by community volunteers yields more TT cases while dealing with less number of patients resulting in better efficiencies. This approach could be used in settings where the number of TT cases is declining. It is however important to note that the method of case finding and the type of outreach should be determined by the context and the system within which the services are delivered.

## Figures and Tables

**Table 1:** Findings from TT case finding in the communities.

Approach	Population of Study Area	# of Suspected TT Cases on CF Registers (% of Population)	# of Suspected TT Cases Presenting to Outreach (% in CF Registers)	# of Suspected TT Cases Confirmed to Have TT (%)
Volunteer case finders (#1)	152,871	736 (0.48)	230 (31.2)	106 (46.1)
Volunteer case finders + VA testing at outreach (#2)	244,234	1276 (0.52)	477 (37.4)	141 (29.7)
Community health worker case finders + VA testing in community (#3)	220,872	1837 (0.83)	853 (46.4)	135 (15.8)
Town criers only (#4)	191,517	NA	NA	NA

#—Number. NA = not applicable. Approach #4 did not include case finding in the communities.

**Table 2:** Gender differences in TT outreach.

Approach	Total # of People Presenting to Outreach	# Men (%)	# Women (%)	# of Confirmed TT Cases	# of Confirmed Cases Having Surgery (%)	# Men (%)	# Women (%)
Volunteer case finders (#1)	403	121 (30.0)	282 (70.0)	131 (32.5)	107 (81.7)	22 (20.6)	85 (79.4)
Volunteer case finders + VA testing at outreach (#2)	1072	429 (40.0)	643 (60.0)	172 (16.0)	149 (86.6)	22 (14.8)	127 (85.2)
Community health worker case finders + VA testing in community (#3)	1419	661 (46.5)	758 (53.5)	169 (11.9)	154 (91.1)	27 (17.5)	127 (82.5)
Town criers only (#4)	1901	744 (39.1)	1157 (60.9)	195 (10.3)	176 (90.3)	28 (15.9)	148 (84.1)

**Table 3:** Productivity of outreach according to approach.

Approach	Total # People Presenting to Outreach (% of Total Population)	# Suspected TT Cases Presenting to Outreach (%)	# People Self-Presenting (%)	# Confirmed TT Cases (% among Presenting to Outreach)	# Confirmed TT among Self-Presenting (%)	Cost of Finding 1 TT Case
Volunteer case finders (#1)	403 (0.26)	230 (57.0)	173 (43.0)	131 (32.5)	25 (14.4)	USD 6.3
Volunteer case finders + VA testing at outreach (#2)	1072 (0.44)	477 (44.5)	595 (55.5)	172 (16.0)	31 (5.1)	USD 5.8
Community health worker case finders + VA testing in community (#3)	1419 (0.64)	853 (60.0)	566 (40.0)	169 (11.9)	34 (6.1)	USD 5.4
Town criers only (#4)	1901 (0.99)	NA	1901 (100)	195 (10.3)	195 (10.3)	USD 21.5

NA: Case finding not undertaken in method 4; column 1% derived using total population found in Table 1.

## Data Availability

The data presented in this study are available on request from the corresponding author.
